# Prolonged fixed dose rate infusion of gemcitabine with autologous haemopoietic support in advanced pancreatic adenocarcinoma

**DOI:** 10.1038/sj.bjc.6602673

**Published:** 2005-06-28

**Authors:** C Bengala, V Guarneri, E Giovannetti, M Lencioni, E Fontana, V Mey, A Fontana, U Boggi, M Del Chiaro, R Danesi, S Ricci, F Mosca, M Del Tacca, P F Conte

**Affiliations:** 1 Department of Oncology and Hematology, Division of Medical Oncology, University of Modena and Reggio Emilia, Via del Pozzo71, Modena 41100, Modena, Italy; 2 Division of Pharmacology and Chemotherapy, University Hospital, Pisa, Italy; 3 Division of Medical Oncology, University Hospital, Pisa, Italy; 4 Division of Surgery, University Hospital, Pisa, Italy

**Keywords:** cytidine deaminase, fixed dose rate infusion, gemcitabine pharmacokinetic, pancreatic cancer

## Abstract

This study aimed to define the maximum-tolerated dose (MTD) of fixed dose rate (FDR) of gemcitabine (2′-2′-difluorodeoxycitidine) infusion with circulating haemopoietic progenitor support and to evaluate the activity of the treatment. Secondary end points were pharmacokinetic of gemcitabine and difluorodeoxyuridina (dFdU) measured at first course and the activity andexpression profile of cytidine deaminase (CdA) on circulating mononuclear cells. Patients with advanced pancreatic carcinoma received escalating dose of gemcitabine 10 mg m^−2^ min^−1^ every 2 weeks with circulating haemopoietic progenitor support. First dose level was 3000 mg m^−2^ and the doses were increased by 500 mg m^−2^ until MTD. In all, 23 patients were enrolled. Toxicities were mild or moderate; the only patient treated at 7000 mg m^−2^ died because of toxicity; therefore; the MTD was established at 6500 mg m^−2^. The overall response rate was 22.2%. The AUC of gemcitabine showed a dose-dependent increase, while the AUC of dFdU reached a plateau at 4500 mg m^−2^. A significant relationship was found between the AUC of dFdU and CdA expression and activity (*P*<0.05). Moreover, progression rate and survival were significantly related to CdA expression and activity levels. The activity of high-dose gemcitabine is not superior to that reported with less intensive FDR schedules. The predictive role of CdA expression and activity on outcome deserves further investigation.

Pancreatic carcinoma is the fifth most common cause of cancer death in Western countries. At diagnosis, the majority of patients have locally advanced unresectable or metastatic disease for which there is no curative therapy (Evans *et al*, 1997, p 1054). For many years, 5-fluorouracil has been considered the standard treatment of pancreatic cancer with response rates ranging from 0 to 19% (Palmer *et al*, 1994). More recently, gemcitabine (2′-2′-difluorodeoxycitidine) has proven activity against a variety of solid tumours including pancreatic cancer (Huang *et al*, 1991; Rothenberg *et al*, 1996). A weekly short time (30 min) intravenous (i.v.) infusion of 1000 mg m^−2^ is the recommended dose and schedule. However, even if effective, this schedule might not to be the optimal one taking into account the metabolism of the drug.

Gemcitabine is a prodrug that is initially phosphorylated by deoxycytidine kinase (dCK) to gemcitabine monophosphate and subsequently to gemcitabine diphosphate and gemcitabine triphosphate. Incorporation of gemcitabine triphosphate into DNA inhibits replication with induction of apoptosis. Phosphorylation of gemcitabine to the monophosphate by deoxycytidine kinase is the rate-limiting step in the accumulation of the active diphosphate and triphosphate metabolites. Grunewald *et al* and Abbruzzese *et al* have demonstrated that the ability of mononuclear cells to accumulate gemcitabine triphosphate during therapy was saturable and the optimal plasma concentration of gemcitabine that maximised the rate of formation of gemcitabine triphosphate was approximately 20 *μ*mol l^−1^ (Grunewald *et al*, 1990, 1991). In these studies, this target plasma gemcitabine concentration and the optimal intracellular accumulation of gemcitabine triphosphate was achieved with dose rates of 10 mg m^−2^ min^−1^ (Abruzzese *et al*, 1991; Grunewald *et al*, 1992). Based on these data, a number of phase I trials have explored the possibility to prolong the duration of infusion time; other trials escalated both the dose and infusion duration using a fixed dose rate (FDR) of 10 mg m^−2^ min^−1^ (Abbruzzese *et al*, 1991; Grunewald *et al*, 1992; Poplin *et al*, 1992; Pollera *et al*, 1997). Tempero *et al* have compared in a phase II randomised trial the standard gemcitabine infusion in 30 min infusion *vs* an FDR infusion of 10 mg m^−2^ min^−1^ in pancreatic carcinoma. A significant increase in response rate, a longer median survival and a higher 1-year survival rate was reported in the FDR infusion-treated patients. However, patients receiving the FDR infusion experienced consistently more haematologic toxicity and cumulative myelosuppression was reported (Tempero *et al*, 2003).

On the basis of these data from this trial and preclinical data suggesting a dose–response relationship (Csoka *et al*, 1995; Von Hoff, 1996), we have designed a dose-finding phase I trial of gemcitabine given as prolonged FDR (10 mg m^−2^ min^−1^) infusion every 14 days with haemopoietic progenitor support in patients with advanced pancreatic adenocarcinoma.

End points of the study were to define the maximum-tolerated dose (MTD) of prolonged FDR of gemcitabine plus progenitor blood cells and to evaluate the activity of the treatment in terms of objective responses. Additive end points of the trial were to study the plasma pharmacokinetic of gemcitabine and its metabolite difluorodeoxyuridina (dFdU) following FDR infusion and to evaluate the influence of gene expression and activity of cytidine deaminase (CdA) on clinical outcome.

## MATERIALS AND METHODS

### Eligibility

Patients ⩽75 years old, performance status 0–1 (ECOG scale) with histologically confirmed unresectable advanced or metastatic pancreatic carcinoma were eligible for the study. Other eligibility criteria were bidimensionally measurable disease, leucocyte count ⩾3.5 × 10^9^ l^−1^, platelet count ⩾100 × 10^3^ l^−1^, haemoglobin level ⩾10 g l^−1^, no active infection, no severe concurrent medical condition and no previous treatment for the advanced disease. Prior adjuvant chemotherapy and/or radiotherapy was permitted. The patients were required to sign an informed consent and the Ethics Committee of St Chiara University Hospital approved the study.

### Treatment plan

Granulocyte-colony-stimulating factor (G-CSF) 10 *μ*g kg^−1^ day^−1^ subcutaneously (s.c.) was administered for 5 days to mobilise circulating haemopoietic progenitor cells. When the peripheral blood absolute CD34+ cell count was ⩾50 *μ*l^−1^, the aphaeresis procedure was performed using a Fenwall CS 3000 cell separator (Baxter, Chicago, IL, USA). Whole blood (8–10 l) was processed per procedure using continuous flow blood at a flow rate of 50 ml min^−1^. A minimum of 12 × 10^6^ CD34+ cell kg^−1^ were collected from each patient.

Gemcitabine was administered at an FDR of 10 mg m^−2^ min^−1^ at the starting dose of 3000 mg m^−2^ (starting time infusion 300 min). If no dose-limiting toxicity occurred in the first two courses, the dose of gemcitabine was escalated by 500 mg m^−2^ in cohorts of three patients until maximum tolerable dose. In the absence of toxicity or progression, the courses were repeated every 14 days for a maximum of 12 courses. Peripheral blood progenitors were reinfused 24 h after each course of chemotherapy. At the time of reinfusion, frozen PBPC were thawed rapidly in a 37°C warm bath and reinfused through a central venous catheter: at least 0.5 × 10^6^ CD34+ cell kg^−1^ were reinfused per course. Following PBPC infusion, G-CSF 5 *μ*g kg^−1^ day^−^1 s.c. was administered starting day +5 to day +7.

### Safety and activity assessment

A complete restaging of disease was performed after four, eight and 12 courses of treatment. Definition of response and toxicity were based on WHO criteria. Dose-limiting toxicity of gemcitabine was defined by one of the following criteria: G3 stomatitis lasting more than 5 days, G4 stomatitis and impossibility to recycle on day 14 in more than 40% of the courses. The MTD of gemcitabine was considered the dose level immediately below that producing one DLT out of three patients.

### Pharmacokinetic analysis

Blood samples (4 ml each) were drawn at the first cycle of gemcitabine administration from an i.v. cannula placed in an antecubital vein before drug administration, after 30 min and every 3 h during infusion and 5, 15, 30, 45 min and 1, 3, 9, 15 and 24 h after the end of treatment and collected in heparinised test tubes containing 10 *μ*M tetrahydrouridine (Calbiochem, San Diego, CA, USA) to inhibit gemcitabine metabolism by CdA. Plasma was obtained by centrifugation at 5000 **g** for 10 min, split in aliquots and stored at −80°C.

Plasma levels of gemcitabine and its metabolite 2′, 2′-difluorodeoxyuridine (dFdU) were determined by a previously described reverse-phase high-performance liquid chromatography with ultraviolet detection (HPLC-UV) method optimised for the present study (Wang *et al*, 2003). Drug extraction was performed by adding 80 *μ*l of buffer (0.1 mol l^−1^ Tris-HCl, 50 *μ*mol l^−1^ *β*-mercaptoethanol, pH 8.0) and 50 *μ*l of 40% (w v^−1^) trichloroacetic acid (TCA) to 120 *μ*l of plasma. Samples were kept on ice for 20 min and proteins were precipitated by centrifugation for 10 min at 10 000 **g**. Supernatants were neutralised with 500 *μ*l trioctylamine : 1,1,2-trichloro-trifluoroethane (1 : 4), centrifuged for 1 min at 10 000 **g** and 50 *μ*l were injected onto a Simmetry Shield C_18_ 5 *μ*m, 300 × 4.6 mm column (Waters, Milford, MA, USA) eluted at a flow rate of 1 ml min^−1^ with 9% methanol, 6% acetonitrile and heptane sulphonic acid 5 mM, pH 3.1. The chromatographic apparatus was a Waters LC Module I plus equipped with a WISP 416 autosampler and a variable wavelength UV detector (Waters) set at 270 nm for peak detection. Calculation of gemcitabine and dFdU concentration in samples was performed against calibration curves in the range of 0.3–300 *μ*mol l^−1^ in the plasma of healthy donors. Maximum plasma concentration (*C*_max_, *μ*mol l^−1^) of gemcitabine and dFdU were determined from visual inspection of the plasma concentration–time curves, while the area under the plasma concentration–time curve (AUC, min × *μ*mol l^−1^) was calculated with the linear trapezoidal rule.

### Pharmacogenomic analysis

#### Analysis of CdA enzyme activity

Mononuclear cells were obtained by density-gradient centrifugation of patients’ blood samples (14 ml), obtained before drug administration, on Ficoll–Hypaque (density 1.077 g l^−1^, Pharmacia, Uppsala, Sweden). Cells were resuspended at 3 × 10^6^ cells 100 *μ*l^−1^ of buffer (0.1 mol l^−1^ Tris-HCl, 50 *μ*mol l^−1^ *β*-mercaptoethanol, pH 8.0 (van Haperen *et al*, 1996). The suspension was sonicated and centrifuged for 20 min at 10 000 **g** to obtain crude cytoplasmatic extracts and 10 *μ*l of 0.5 mmol l^−1^ gemcitabine was added to 50 *μ*l of supernatant and 40 *μ*l of buffer. Reaction mixture was incubated for 30 min at 37°C and extraction of analytes was obtained with 25 *μ*l 40% TCA. Samples were kept on ice for 20 min, neutralised with 250 *μ*l tryoctylamine : 1,1,2-trichloro-trifluoroethane (1 : 4) and centrifuged for 5 min at 10 000 **g**. The upper layer was removed and analytes were separated by HPLC, as described above in ‘Pharamacokinetic analysis’. Human mononuclear cells from blood donors were the source of cytoplasmatic extracts to be used as a calibrate matrix and spiked with dFdU to generate calibration curves, in the range of 33–1000 *μ*mol l^−1^. The HPLC method was linear (*R*^2^>0.995) for plasma and cytoplasmatic extracts over the analytical range and the limit of quantitation corresponded to the lower limit of the calibration curves. Intraday variability was >−2.5 and <12% over the full analytical range of calibration standards, while interday variability was >−1.5 and <15%. Concentrations of dFdU were normalised for cytoplasm protein concentration, which was measured with the Lowry reagent (Sigma, St Louis, MO, USA).

#### PCR analysis of CdA gene expression

Total RNA was extracted using the TRI REAGENT LS (Sigma, St Louis, MO, USA) and dissolved in RNAse-free water containing 10 mmol l^−1^ dithiothreitol and 200 U ml^−1^ RNase inhibitor (Gibco, Gaithersburg, MD, USA). RNA (1 *μ*g) was reverse transcribed at 37°C for 1 h in 100-*μ*l reaction volume containing 0.8 mM dNTPs (Sigma, St Louis, MO, USA), 200 U of MMLV-RT (Gibco, Gaithersburg, MD, USA), 40 U of RNase inhibitor and 0.05 *μ*g ml^−1^ of random primers. The cDNA was amplified by quantitative, real-time PCR with the Applied Biosystems 7900HT sequence detection system (Applied Biosystems, Foster City, CA, USA). PCR reactions were performed in triplicate using 5 *μ*l of cDNA, 12.5 *μ*l of TaqMan Universal PCR Master Mix (Applied Biosystems), 2.5 *μ*l of probe and 2.5 *μ*l of forward and reverse primers in a final volume of 25 *μ*l. Forward (F) and reverse (R) primers and probe (P) were designed with Primer Express 2.0 (Applied Biosystems) on the basis of CdA gene sequence: 5′-TCAAAGGGTGCAACATAGAAAATG-3′ (F), 5′-CGGTCCGTTCAGCACAGAT-3′ (R) and 5′-CTGCTACCCGCTGGG-3′ (P) (Demontis *et al*, 1998). PCR thermal cycling conditions and optimisation of primer concentrations were reported in detail by Giovannetti *et al* (2004). The amount of target gene, normalised to GAPDH and relative to the calibrator (mononuclear cells from volunteer blood donors), is given as 2^−ΔΔ*C*_T_^

.

### Statistics

Data were expressed as mean values±s.d. and the Pearson/Spearman correlation test and regression analysis were used to demonstrate the relationship between CdA expression, enzyme activity and dFdU AUC. Correlation between response rate and CdA expression and activity level was performed using *χ*^2^ test. Overall survival (OS) curves were calculated from the date of diagnosis until the date of death or last follow-up examination according to the Kaplan–Meier method and compared by the log-rank test. Variables studied in univariate analysis included CdA expression and activity levels, dFdU AUC, age, sex and drug dose levels. Differences were considered significant if the *P*<0.05.

## RESULTS

### Characteristics of the patients

In all, 25 patients with locally advanced or metastatic pancreatic carcinoma entered into this the study. Two patients were unable to receive the planned treatment due to poor mobilisation of peripheral blood progenitors. A total of 23 patients collected the minimum number of CD34+ cells to support at least one course of treatment. The median age was 56 years (range 42–75 years) and median performance status (WHO scale) was 0 (range 0–1). Three patients had locally advanced disease, 20 had metastatic disease: two patients had received previous adjuvant chemotherapy with gemcitabine as single agent. Predominant metastatic site was the liver in 14 patients (60.9%) (Table 1). The median number of aphaeresis procedure per patients was 3 (range 2–7) and median number of CD34+ cell kg^−1^ collected per aphaeresis was 2.23 × 10^6^ (range 0.15–10.1). Owing to the poor collection after G-CSF only, 15 patients underwent a further mobilisation with gemcitabine+G-CSF. The average of CD34+ cell kg^−1^ collected per aphaeresis after G-CSF and after gemcitabine+G-CSF was 2.18 × 10^6^ (s.d.±1.65 × 10^6^) and 4.12 × 10^6^ (s.d.±2.46 × 10^6^), respectively. The difference was statistically significant (*P*=0.0001).

### Toxicity

Three patients were treated for each dose level from 1st (3000 mg m^−2^ in 300 min) to 6th (6000 mg m^−2^ in 600 min), four patients at 7th dose level (6500 mg m^−2^ in 650 min) and one patient at 8th dose level. A total of 137 courses were administered and were evaluable for toxicity. In all, 15 courses were administered at 1st level, 19 at 2nd, 16 at 3rd, 15 at 4th, 32 at 5th, 27 at 6th, 13 at 7th and 1 at 8th. G3–4 nonfebrile neutropenia occurred in 2.8% of courses, G3 anaemia in 6.5% of courses and G3 thrombocytopenia in 1.5% of courses. The patient treated at 7000 mg m^−2^ experienced a G4 mucositis and died 25 days after the first course of treatment. Mucositis was considered the dose-limiting toxicity and 6500 mg m^−2^ was defined as the MTD. Other haematologic and nonhaematologic toxicities were mild or moderate: G2 fever was reported in 11.6% of courses, G1 dermatitis in 6.4% and G1 deep venous thrombosis in 2.9% (Table 2).

### Outcome

**Figure 1:**
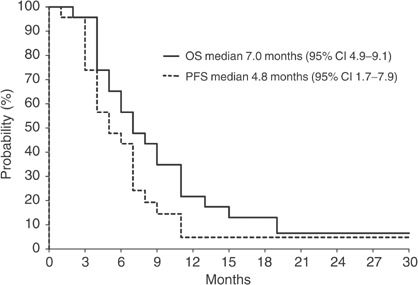
Progression-free survival (PFS) and overall survival (OS) of 23 patients.

In all, 18 patients completed at least four courses of treatment and are evaluable for response. Four patients (22.2%) achieved an objective response: one patient achieved a complete remission lasting 21+ months and three patients achieved a partial remission; seven patients (36.8%) had a stable disease. Seven patients experienced early progression. Five patients received less than four courses of chemotherapy due to refusal (two patients) and DVT (three patients). According to the intention-to-treat analysis, median time to progression was 4.8 months (95% CI 1.7–7.9) and median OS was 7.0 months (95% CI 4.9–9.1); the 1-year survival rate was 21.9%. (Figure 1).

#### Pharmacokinetic analysis

The plasma concentration of gemcitabine reached the target concentration of 15 *μ*mol l^−1^ within 30 min from the start of infusion and remained above this threshold level during the infusion period. The maximum plasma concentration (*C*_max_) of gemcitabine was achieved at the end of the infusion and ranged from 24.84±3.93 to 84.36±15.97 *μ*mol l^−1^, suggesting that there was heterogeneity in the plasma kinetics among the patients, whereas the AUC ranged from 5588.72±1447.85 to 33402.95±3136.62 min × *μ*mol l^−1^, showing a dose-dependent increase. Gemcitabine rapidly disappeared from plasma, and 30 min after the end of drug infusion, only the metabolite dFdU was detectable. *C*_max_ of dFdU ranged from 77.51±8.70 to 144.431±27.79 *μ*mol l^−1^ and was achieved approximately 15 min after the end of gemcitabine infusion. The AUC ranged from 36 668.76±3153.76 to 51 314.08±8958.63 min × *μ*mol l^−1^, corresponding to the 3000–4500 mg m^−2^ dose levels; over the 4500 mg m^−2^ dose, the AUC of the metabolite did not increase, possibly depending on saturable metabolism (Table 3).

#### Correlation between CdA gene expression, enzyme activity and dFdU pharmacokinetics

**Figure 2:**
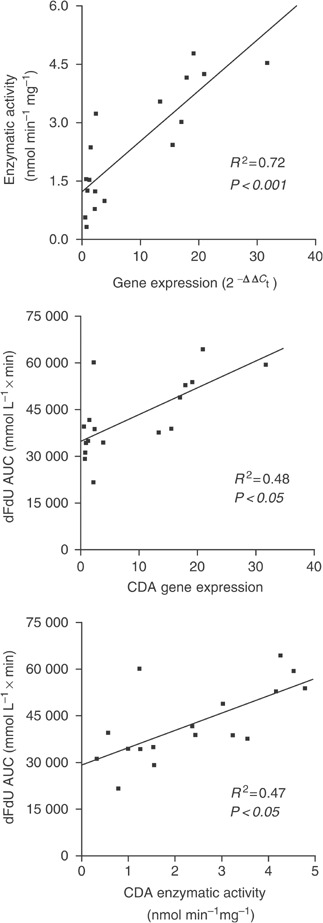
Relation between CDA mRNA expression, enzymatic activity and the AUC of dFdU.

CdA gene expression in mononuclear cells from patients showed a 57-fold variation among patients, ranging from 0.56 to 31.67; in addition, the enzymatic activity varied from 0.32 to 4.78 nmol min^−1^ mg^−1^ protein. A significant relationship was found between CdA expression and activity (*R*^2^=0.72, *P*<0.001), as well as between the plasma AUC of dFdU and CdA expression and its activity (*P*<0.05) (Figure 2).

#### Relationship between CdA expression, CdA activity and clinical outcome

**Figure 3:**
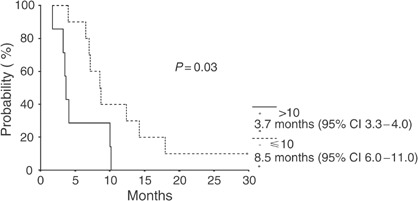
Overall survival analysis according to CdA expression levels: ⩽10 (10 patients) and >10 (seven patients).

**Figure 4:**
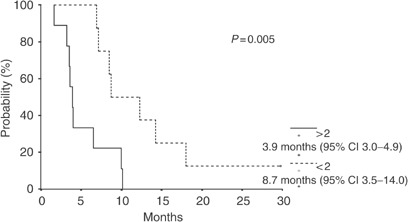
Overall survival analysis according to CdA activity levels: ^−1^ mg^−1^ (eight patients) and >2 nmol min^−1^ mg^−1^ (nine patients).

The analysis of CdA expression and activity was performed on 17 patients and 16 of these are evaluable for response. Patients with mononuclear cells’ CdA expression level <10 U and CdA activity level <2 nmol min^−1^ mg^−1^ protein achieved an objective response (one CR, three PR) or a stable disease (six patients). On the contrary, patients with CdA expression level >10 U and CdA activity level >2 nmol min^−1^ mg^−1^ protein showed an early progression of disease (six patients): the differences are statistically significant with *P*<0.05. In a univariate analysis, patients whose mononuclear cells had CdA expression levels >10 (seven patients, median survival time, 3.65 months, 95% CI 3.31–3.98) had significantly (*P*=0.03) shorter OS than patients with CdA expression levels <10 (10 patients, median survival time, 8.51 months, 95% CI 6.01–11.00) (Figure 3). Similar results were obtained with CdA activity analysis; patients whose mononuclear cells had CdA activity levels >2 nmol min^−1^ mg^−1^ protein (nine patients, median survival time, 3.98 months, 95% CI 3.02–4.93) had significantly (*P*=0.006) shorter OS than patients with CdA activity levels <2 nmol min^−1^ mg^−1^ protein (eight patients, median survival time, 8.74 months, 95% CI 3.46–14.02) (Figure 4). In contrast, no significant impact on OS was found both for dFdU AUC <40.000 min × *μ*mol l^−1^ and for dose level >4500 mg m^−2^. Finally, among the other parameters studied, male sex was associated with shorter survival (median survival time, 5.22 months, 95% CI 1.75–8.70, *vs* 10.02 months, 95% CI 3.20–16.84, *P*=0.04), while no significant impact on OS was found for age (<56 years).

## DISCUSSION

The MTD of gemcitabine is strongly associated to the infusion schedule: when administered daily for 5 days over 30 min, the MTD was reached at 9 mg m^−2^ day^−1^ (O’Rourke *et al*, 1994); on the other hand, when the drug was administered biweekly, the MTD was observed at 4000 mg m^−2^ day^−1^ (Brown *et al*, 1991). In the pivotal trial conducted by Burris *et al* (1997), gemcitabine produced a response rate of 5.4%, a median survival time of 5.7 months and the clinical benefit response in 23.8% of the patients. In this trial, gemcitabine was administered on a weekly basis as a 30-min infusion and this schedule has become the standard modality of administration. In a phase I trial, when gemcitabine was administered at FDR to patients with previously treated solid tumours including pancreatic cancer, the MTDs were 1500 mg m^−2^ over 150 min (Brand *et al*, 1997) and 1800 mg m^−2^ over 180 min weekly for 3 weeks (Touroutoglou *et al*, 1998). Preclinical and clinical data suggest that an infusion rate of 10 mg m^−2^ min^−1^ may be more effective even if myelotoxicity can become the dose-limiting toxicity (Veerman *et al*, 1996; Tempero *et al*, 2003). An FDR of 10 mg m^−2^ min^−1^ can provide plasma concentrations (approximately 20 *μ*mol l^−1^) that are sufficient to maximise the rate of gemcitabine triphosphate accumulation. Two phase I trials have shown that a single course of gemcitabine as FDR infusion of 10 mg m^−2^ min^−1^ for 12 h can be safely administered alone or in combination with mitoxantrone in patients with relapsed/refractory acute leukaemia (Gandhi *et al*, 2002; Rizzieri *et al*, 2002).

The data of our study demonstrate that multiple courses of gemcitabine can be administered at the FDR of 10 mg m^−2^ min^−1^ up to 11 h every 14 days with peripheral blood stem cell support. Tolerability was excellent with a G4 neutropenia observed in 1.4% of the courses only, while other haematological and clinical toxicities were ⩽grade 3. All the courses were administered at the scheduled 2-week interval. Surprisingly, the first patient treated at the dose level of 7000 mg m^−2^ developed a severe mucositis and died at day 25 after the first course. It was therefore decided to stop the dose escalation in spite of excellent tolerability experienced by all other patients.

Unfortunately, the activity of this intensive treatment was lower than expected: an objective response was observed in 22.2% of patients, while a disease control (OR+s.d.) was achieved in 61% of the patients; median TTP and OS were 4.8 and 7 months, respectively, and 1 year survival rate was 21.9%. These results are inferior to those reported by Tempero *et al* (2003) with gemcitabine given at FDR of 1500 mg m^−2^ without peripheral blood stem cell support.

In agreement with Tempero *et al*, the mean plasma gemcitabine concentration at the end of an FDR infusion of 10 mg m^−2^ min^−1^ was lower than that of the standard 30-min infusion. Conversely, *C*_max_ of gemcitabine ranged from 24.84±3.93 to 84.36±15.97 *μ*mol l^−1^, while in a standard 30-min infusion with a dose level of 2000 mg m^−2^, the *C*_max_ was 136.17±49.50 *μ*mol l^−1^ (De Pas *et al*, 2000). However, the FDR infusion was able to generate sustained gemcitabine levels in plasma above the threshold of 15 *μ*mol l^−1^, which is higher than the concentration required to achieve maximal intracellular accumulation of gemcitabine triphosphate; moreover, the gemcitabine AUC showed a dose-dependent increase. In contrast, the AUC of the gemcitabine metabolite showed a dose-dependent increase until the 4500 mg m^−2^ dose levels; above this dose level, the AUC did not increase, possibly depending on saturable metabolism.

The data of the present study also demonstrate a genotype–phenotype correlation between CdA gene expression, enzyme activity and AUC of the metabolite dFdU produced by gemcitabine deamination by CdA. These findings are in agreement with a previous study by Schroeder *et al* that demonstrated a significant correlation between the amount of CdA mRNA and enzyme activity in leukaemic blasts, suggesting that variations in CdA activity depends on differences in gene expression. Schroder *et al* (1998) also showed a correlation of pretherapeutic CdA activity with induction treatment response with the nucleoside analogue cytarabine in patients with acute myeloid leukaemia. Kroep and co-workers have recently shown a clear correlation between dCK levels and gemcitabine sensitivity. In contrast to dCK, CDA activity was not clearly related to gemcitabine sensitivity (Kroep *et al*, 2002).

In the present study, an univariate analysis demonstrated that patients whose mononuclear cells had higher CdA expression and activity levels had significantly higher rate of early progression and shorter OS than patients with lower CdA expression and activity levels, suggesting that gemcitabine metabolism in mononuclear cells could predict clinical outcome. The small number of patients in our study does not allow a multivariate analysis.

In conclusion, gemcitabine given as an FDR infusion of 10 mg m^−2^ min^−1^ in pancreatic cancer patients is feasible with peripheral blood cell support up to 650 min and the dose of 6500 mg m^−2^ is the MTD. No severe toxicities were observed at this dose; however, the activity does not seem to be better than that reported with other less intensive FDR schedules. Pharmacokinetic data demonstrate a correlation between dose of gemcitabine and its AUC; on the contrary, AUC of dFdU showed a plateau over 4500 mg m^−2^ of gemcitabine, possibly depending on saturable metabolism. The observed relationship between CdA expression and activity in mononuclear cells and treatment activity if confirmed might be useful to discriminate patients at different prognosis.

## Figures and Tables

**Table 1:** Patient characteristics (range)

No. of patients	23
M/F	13/10
Median age (years)	56 (42–75)
Median ECOG PS	0 (0–1)
Locally advanced disease (no. of pts.)	3
Metastatic disease (no. of pts.)	20
Previous adjuvant CT (no. of pts.)	2
Median no. of aphaeresis/pt.	3 (2–7)
Median CD34+ cells kg^−1^/aphaeresis	2.23 × 10^6^ (0.15–10.1)
G-CSF	1.79 × 10^6^ (0.15–9.12)
Gemcitabine+G-CSF	3.61 × 10^6^ (0.89–10.1)
No. of total courses	137

ECOG PS=Eastern Cooperative Oncology Group Performance Status; G-CSF=granulocyte-colony-stimulating factor.

**Table 2:** Toxicities (WHO scale) 137 courses

	**G1 (%)**	**G2 (%)**	**G3 (%)**	**G4 (%)**
Anaemia	24 (17.5)	16 (11.6)	9 (6.5)	—
Neutropenia	2 (1.4)	6 (4.3)	2 (1.4)	2 (1.4)
Thrombocytopenia	3 (4.4)	2 (2.9)	1 (1.5)	—
Diarrhoea	—	2 (2.9)	—	—
Fever	—	16 (11.6)	—	—
Dermatitis	5 (3.6)	—	—	

WHO=World Health Organization.

**Table 3:** Plasma pharmacokinetics of gemcitabine and dFdU

	**Gemcitabine**		**DfdU**	
**Dose (mg m^−2^)**	***C*_max_ (*μ*mol l^−1^)**	**AUC (min × *μ*mol l^−1^)**	***C*_max_ (*μ*mol ml^−1^)**	**AUC (min × *μ*mol l^−1^)**
3000	24.84±3.93	5588.72±1447.85	77.51±8.70	38 026.64±16 537.09
3500	35.98±5.52	7700.69±530.94	108.50±4.87	36 879.94±2704.15
4000	44.39±6.34	11 914.78±1462.83	136.44±17.32	51 039.50±2573.54
4500	58.04±13.40	17 100.97±2650.02	144.43±27.79	51 314.08±8958.63
5000	84.36±15.97	28 782.27±4597.91	141.85±57.59	35 398.34±5910.78
6000	76.52±6.45	31 119.09±1971.54	109.613±15.24	41 235.61±16 601.49
6500	74.77±6.93	33 402.95±3136.62	110.12±14.62	36 668.76±3153.76
